# LncRNA regulates tomato fruit cracking by coordinating gene expression via a hormone-redox-cell wall network

**DOI:** 10.1186/s12870-020-02373-9

**Published:** 2020-04-15

**Authors:** Lingzi Xue, Mintao Sun, Zhen Wu, Lu Yu, Qinghui Yu, Yaping Tang, Fangling Jiang

**Affiliations:** 1 College of Horticulture, Nanjing Agricultural University, Weigang NO 1, Nanjing, 210095 Xuanwu District China; 2 Key Laboratory of Horticultural Plant Biology and Germplasm Innovation in East China, Ministry of Agriculture, Nanjing, 210095 China; 3 Institute of Vegetables and Flowers, Chinese Academy of Agricultural Sciences, Zhongguancun South St, Beijing, 10081 Haidian District China; 4 Institute of Vegetables, Xinjiang Academy of Agricultural Sciences, Nanchang Road 403, Urumchi, 830091 Shayibake District China

**Keywords:** Tomato, LncRNA, mRNA, Transcriptome, Network, Fruit cracking

## Abstract

**Background:**

Fruit cracking occurs easily under unsuitable environmental conditions and is one of the main types of damage that occurs in fruit production. It is widely accepted that plants have developed defence mechanisms and regulatory networks that respond to abiotic stress, which involves perceiving, integrating and responding to stress signals by modulating the expression of related genes. Fruit cracking is also a physiological disease caused by abiotic stress. It has been reported that a single or several genes may regulate fruit cracking. However, almost none of these reports have involved cracking regulatory networks.

**Results:**

Here, RNA expression in 0 h, 8 h and 30 h saturated irrigation-treated fruits from two contrasting tomato genotypes, ‘LA1698’ (cracking-resistant, CR) and ‘LA2683’ (cracking-susceptible, CS), was analysed by mRNA and lncRNA sequencing. The GO pathways of the differentially expressed mRNAs were mainly enriched in the ‘hormone metabolic process’, ‘cell wall organization’, ‘oxidoreductase activity’ and ‘catalytic activity’ categories. According to the gene expression analysis, significantly differentially expressed genes included Solyc02g080530.3 (*Peroxide, POD*), Solyc01g008710.3 (*Mannan endo-1,4-beta-mannosidase, MAN*), Solyc08g077910.3 (*Expanded, EXP*), Solyc09g075330.3 (*Pectinesterase, PE*), Solyc07g055990.3 (*Xyloglucan endotransglucosylase-hydrolase 7, XTH7*), Solyc12g011030.2 (*Xyloglucan endotransglucosylase-hydrolase 9, XTH9*), Solyc10g080210.2 (*Polygalacturonase-2, PG2*), Solyc08g081010.2 (*Gamma-glutamylcysteine synthetase, gamma-GCS*), Solyc09g008720.2 (*Ethylene receptor, ER*), Solyc11g042560.2 (*Ethylene-responsive transcription factor 4, ERF4*) etc. In addition, the lncRNAs (XLOC_16662 and XLOC_033910, etc) regulated the expression of their neighbouring genes, and genes related to tomato cracking were selected to construct a lncRNA-mRNA network influencing tomato cracking.

**Conclusions:**

This study provides insight into the responsive network for water-induced cracking in tomato fruit. Specifically, lncRNAs regulate the hormone-redox-cell wall network, including plant hormone (auxin, ethylene) and ROS (H_2_O_2_) signal transduction and many cell wall-related mRNAs (*EXP, PG, XTH*), as well as some lncRNAs (XLOC_16662 and XLOC_033910, etc.).

## Background

Fruit cracking, one of the main disorders in fruit production, can easily cause adverse impacts in fruit marketability such as reducing fruit quality due to a poor appearance, decreasing shelf life, and even making the fruit unmarketable because of fungal infection [1].

Fruit cracking occurs easily under unsuitable environmental conditions. For instance, under abiotic stress caused by dry to very wet conditions, there will be a rapid flow into the fruit, and if the skin loses strength and elasticity due to factors such as maturation, cracking is most likely to occur [2]. It is widely accepted that plants have developed defence mechanisms and regulatory networks to respond to abiotic stress, which involve perceiving, integrating and responding to stress signals by modulating the expression of related genes [3–8]. Fruit cracking is also a kind of physiological disease caused by abiotic stress. Is there a regulatory network involved in fruit cracking?

Since the 1930s, researchers have performed many theoretical and practical studies on cracking [2, 9, 10]. Cracking is the result of a combination of internal and external factors. The internal factors are the fruit’s own characteristics (fruit size, shape, firmness, deposition of cutin, wax, strength of the pericarp, arrangement of cells in the pericarp, quantity and status of stomata, accumulation of osmoregulatory substances such as soluble sugars, growth stage of the fruit, etc.), while the external factors mainly include environmental factors (humidity, light, temperature, wind, etc.) and cultivation management measures (irrigation, mineral nutrition, plant regulation, etc.) [10–13]. Cortes [14] comprehensively analysed 62 genotypes and found that the correlation of cracked fruit with heredity was significantly greater than that with the environment, indicating that the cracking characteristic can show stable heritability and be regulated by certain genes.

Notably, cell wall components and modifications appear to be correlated with the strength of the skin and fruit cracking [15–18]. As ripening proceeds, cell wall degradation gradually occurs, and the fruit cracking rate increases [15, 16]. The cell wall is composed of a cellulose-hemicellulose (Cel-Hem) network and pectin, which is essential to maintain the mechanical strength of the cell wall. As the fruit matures, enzymes and proteins that degrade the polysaccharide components of the cell wall are produced, such as *polygalacturonase (PG), extended protein (EXP), pectin methylesterase (PME), pectate lyases (PL), pectinase, β-galactosidase. (β-gal) and cellulase (Cx)* [19–22]. The synergistic action of these enzymes leads to the degradation of cell wall polysaccharides and softening of the mature fruit peel [23]. Previous research has shown that genes such as *EXP*, *PG, β-gal* and *XET* are associated with fruit cracking [24–29]. Inhibition of *β-gal* gene expression increases the rate of fruit cracking [27]. In tomato, inhibition of *LePG* expression slightly reduces the rate of fruit cracking [28]. Simultaneous suppression of *SlPG* and *SlEXP1* expression in ripening fruits reduces cell wall disassembly and thereby reduces the fruit cracking rate by approximately 12% [30]. It is not a single gene but many genes working together that regulate fruit cracking [30–33]. It remains unclear whether there are other genes related to fruit cracking and which one is the major gene.

NcRNAs (Non-coding RNA) are involved in a lot of life processes, such as cell growth, differentiation, proliferation, and apoptosis [34–38]. In contrast to the approximately 2% of protein-coding genes, more than 90% of genes do not have the ability to encode proteins and are transcribed into ncRNAs [34]. These ncRNAs were originally thought to represent “expression noise” or “expression waste”, but they have now been proven to be strictly regulated to play important roles in the biological processes of organisms and exhibit extremely complex biological functions [36–38]. While much of the published work on ncRNAs has been conducted in humans and animals, the studies on plants are limited to certain model plants, such as Arabidopsis, maize, and wheat [39, 40]. Xin [39] identified 125 stress-related lncRNAs (long non-coding RNA) in wheat, among which 71 responded to powdery mildew, and 77 responded to heat stress. Swiezewski [40] discovered that cold-induced long antisense intragenic RNA (*COOLAIR*) is involved in the vernalization process and regulates the expression of the plant flowering suppressor “gene flowering locus C” (*FLC*). Wang [41] reported the expression and evolution of lncRNAs in *Solanaceae*. Cui’s [42] results provided insights into the WRKY1 − lncRNA33732 − RBOH module involved in the regulation of H_2_O_2_ accumulation and resistance to pathogens in tomato. Wang [43] identified several lnRNAs that are involved in *TYLCV* infection by virus-induced gene silencing (VIGS) and genome-wide analysis. However, none of these studies have focused on lncRNAs and fruit cracking. Is it possible that lncRNAs play important roles in fruit cracking too?

This study aimed to obtain a global view of the transcriptional regulation (mRNAs and lncRNAs) of fruit cracking induced by irrigation in tomato. Differentially expressed mRNAs and lncRNAs related to fruit cracking were identified through transcriptome profiling and bioinformatic analysis. Finally, we determined a lncRNA-regulated hormone-redox-cell wall network for water-induced cracking in tomato. The findings reported here can increase our understanding of the transcriptional regulatory mechanisms of fruit cracking.

## Results

### RNA sequencing and identification of lncRNA and mRNA

In total, we obtained 0.81 to 1.14 billion raw reads and 0.79 to 1.14 billion clean reads from CR and CS tomatoes at various time points (0 h, 8 h and 30 h of saturated irrigation treatment) **(**Table 1**)**. Through genomic comparison, Cufflinks splicing, and CPC2 and PFAM analysis, we identified 1 annotated lncRNA, 2508 putative lncRNAs, 33,784 annotated mRNAs and 409 novel mRNAs **(**Additional file 1**)**.

**Table 1:** The overall assessment of the sequencing data

Sample_name	Raw_reads	Clean_reads	Raw_bases(G)	Clean_bases(G)	Error rate(%)	Q20(%)	Q30(%)	GC_content(%)
CR_0h_1	102,656,082	102,656,082	15.4	15.4	0.01	98.13	95.03	42.82
CR_30h_1	80,567,758	80,567,758	12.09	12.09	0.02	96.32	91.05	42.29
CR_8h_1	79,478,876	79,478,876	11.92	11.92	0.02	96.41	91.23	42.94
CR_0h_2	97,730,610	97,730,610	14.66	14.66	0.01	97.99	94.69	42.52
CR_30h_2	100,192,938	100,192,938	15.03	15.03	0.01	97.98	94.69	42.44
CR_8h_2	94,324,174	94,324,174	14.15	14.15	0.01	98.1	94.97	42.77
CS_0h_1	95,783,116	95,783,116	14.37	14.37	0.01	97.58	93.81	42.95
CS_30h_1	90,916,088	90,916,088	13.64	13.64	0.02	96.86	92.29	42.84
CS_8h_1	86,534,460	86,534,460	12.98	12.98	0.01	97.79	94.28	42.96
CS_0h_2	103,713,024	103,713,024	15.56	15.56	0.01	98.02	94.78	42.79
CS_30h_2	98,559,578	98,559,578	14.78	14.78	0.01	97.97	94.71	42.39
CS_8h_2	114,298,442	114,298,442	17.14	17.14	0.01	97.89	94.49	43

### Feature analysis of lncRNAs and identification of lncRNA-mRNA pairs

**Fig. 1:**
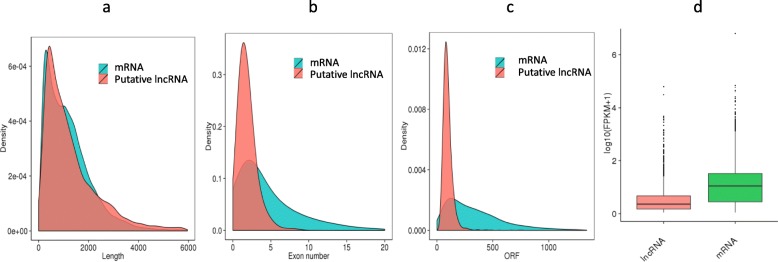
Comparative features of mRNAs and lncRNAs. **a** Length distribution of mRNAs and lncRNAs. **b** Exon number distribution of mRNAs and lncRNAs. **c** ORF length distribution of mRNAs and lncRNAs. **d** Expression levels are indicated as log10 (FPKM + 1) values for the mRNAs and lncRNAs

The average length of the obtained lncRNAs was 1470 nt, which was similar to that of the mRNAs (1221 nt); the average number of exons and average ORF length of the identified lncRNAs were 2.6 and 88.5 bp, which were much lower values than those for the mRNAs (4.7 and 347 bp) (Fig. 1), consistent with previous studies [44, 45]. At the same time, we used phyloP to separately score the lncRNAs and mRNAs, and the sequence conservation of the lncRNAs was lower than that of mRNAs, which was consistent with previous studies [46]. We identified 21,048 lncRNA-mRNA pairs with target relationships upstream and downstream of 2508 lncRNAs (Additional file 2).

### Differential expression analysis

**Fig. 2:**
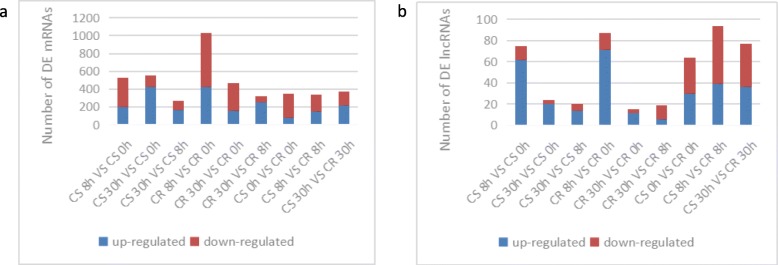
Differentially expressed mRNAs and lncRNAs in different libraries. Upregulated (blue) and downregulated (red) mRNAs and lncRNAs were quantified. **a** mRNAs. **b** lncRNAs. Note: CS is cracking-susceptible, CR is cracking-resistant. The same abbreviations are used below

Differentially expressed mRNAs and lncRNAs were analysed in CR and CS tomatoes by using edge R software **(**Fig. 2**)** and the number of differentially expressed genes was listed in the Additional file 3. mRNAs and lncRNAs with a *Q*-value< 0.05 and |log2 fold-change| > 1 were selected as differentially expressed genes.

### Functional prediction of DEGs

**Fig. 3:**
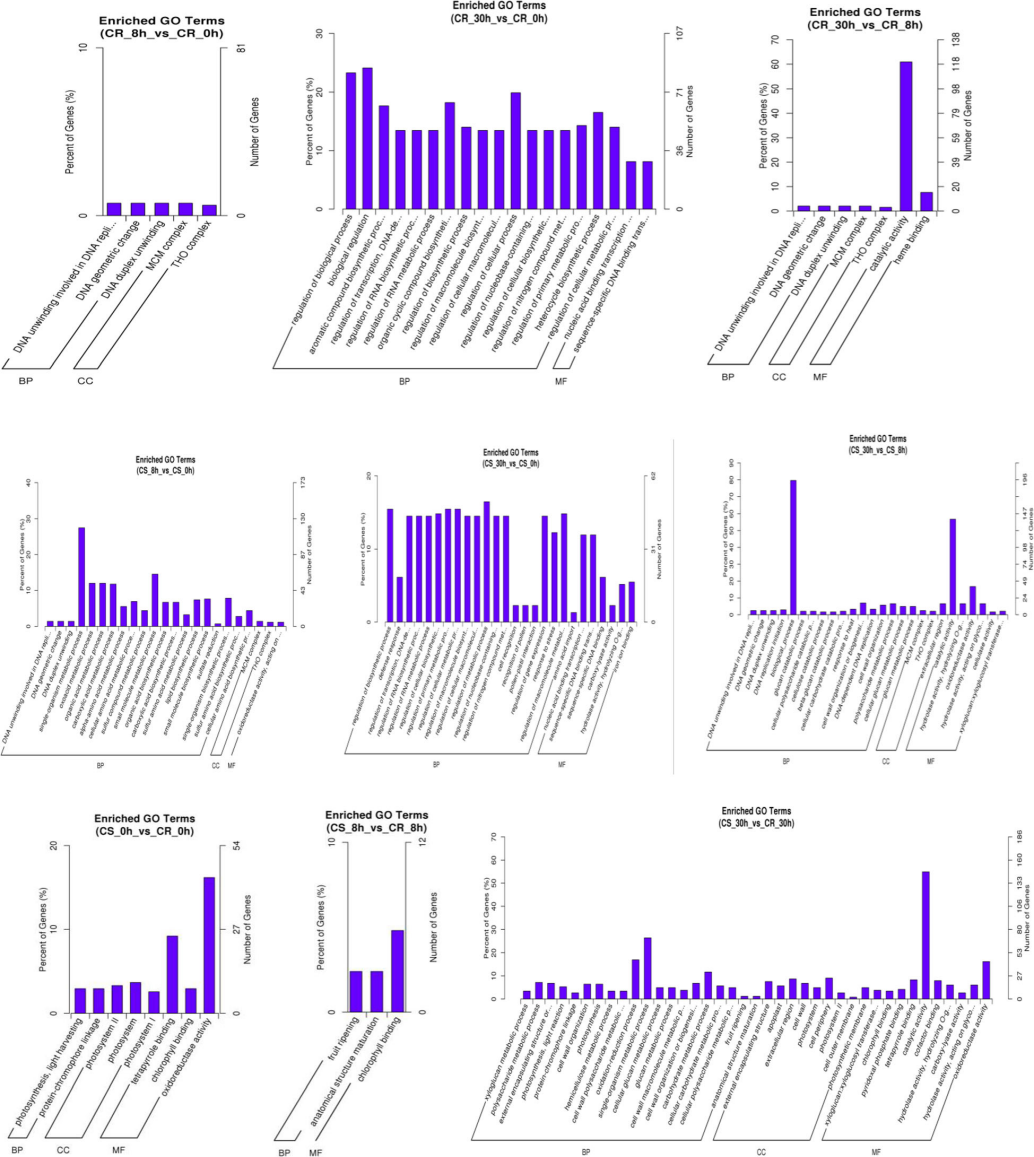
GO classification of transcripts of DEGs. The x-axis indicates number of DEGs, and the y-axis indicates the GO term

To investigate the trends in gene functions and enrichment for DEGs, we performed GO (Gene Ontology) analysis of the selected mRNAs **(**Fig. 3**;** Additional file 4**)**. The results showed that DEGs in the CR tomato were involved in a series of biological processes, such as regulation of biological process, biological regulation and regulation of cellular process, as well as catalytic activity. For the CS tomato, DEGs were mainly involved in single-organism metabolic process, biological process and catalytic activity. Between the CR and CS tomatoes, before irrigation treatment (0 h), DEGs were significantly enriched in oxidoreductase activity; after 8 h of irrigation treatment, there were some DEGs enriched in fruit ripening, anatomical structure maturation, and ageing; after 30 h of irrigation treatment, the number of DEGs enriched in the catalytic category was the highest, followed by the single-organism metabolic process and oxidation-reduction process categories, and cell components.

**Fig. 4:**
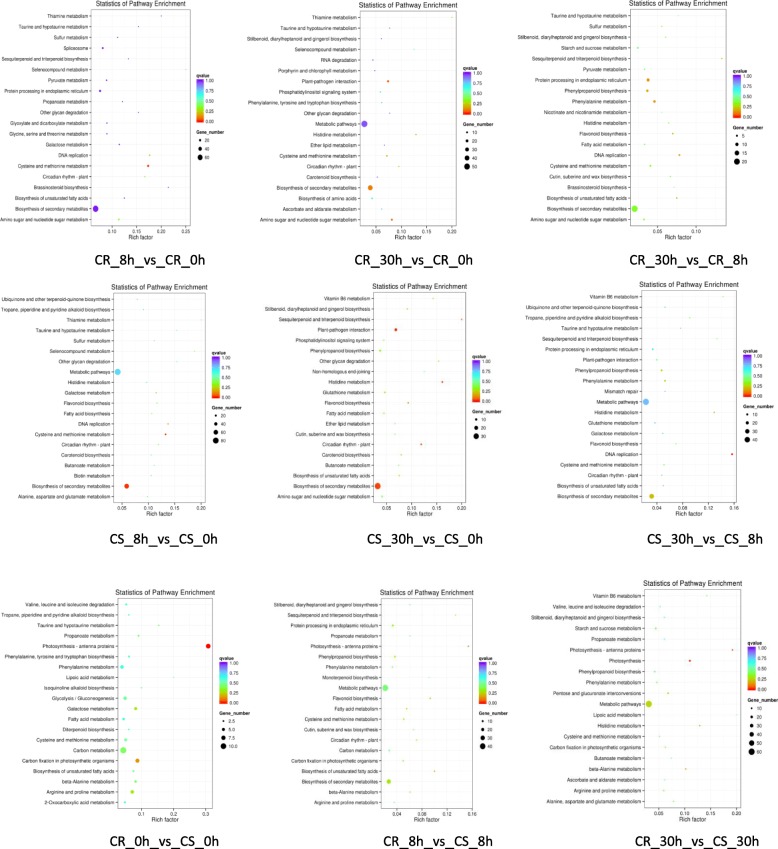
The top 20 KEGG pathways enriched by DEGs. The x-axis indicates the enrichment factor, and the y-axis indicates the pathway names

To better understand the function of the DEGs, significantly enriched KEGG pathways were analysed **(**Fig. 4**;** Additional file 5**)**. The results showed that the DEGs were mainly enriched in the ‘biosynthesis of secondary metabolites’, ‘cysteine and methionine metabolism’, ‘metabolic pathways’, ‘plant-pathogen interaction’, ‘photosynthesis-antenna protein’, ‘photosynthesis’, ‘histidine metabolism’ and ‘circadian rhythm-plant’ categories.

### DEGs (cell wall, redox, hormone related) regulate tomato fruit cracking

**Fig. 5:**
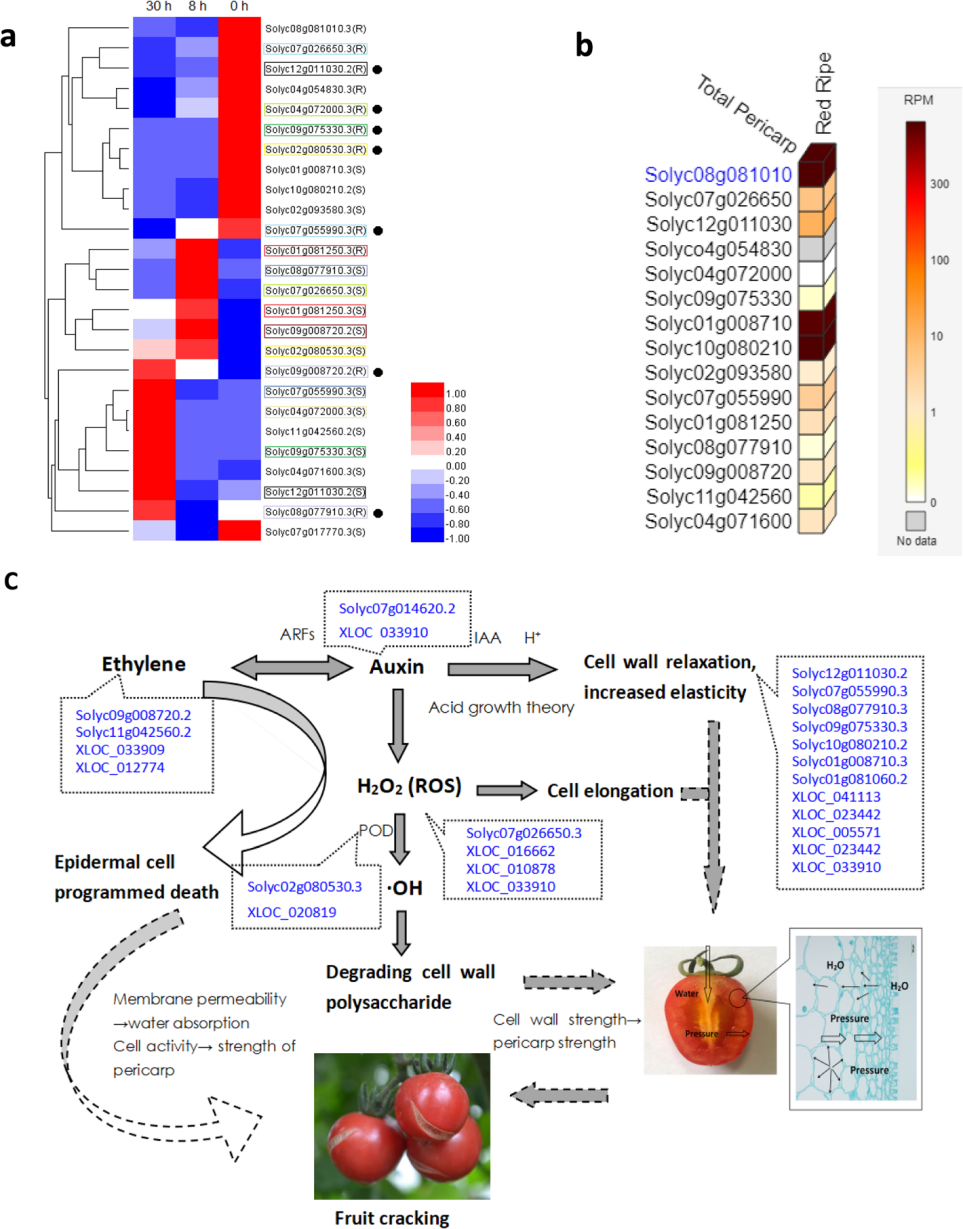
Hierarchical clustering analysis and proposed fruit cracking regulatory network. **a** Hierarchical clustering analysis showed the expression profiles of key genes involved in tomato fruit cracking. Based on the Euclidean distance, the minimum linkage method was used for cluster analysis. Boxes of the same colour represent the same gene in CS and CR. The solid dark circles represent genes that showed different expression trends in the two genotypes. **b** Gene expression cube in total pericarp of tomato during red ripen stage by using Tomato Gene Expression Atlas. **c** Predicted pathway diagram of fruit cracking in tomato, including hormones, reactive oxygen species, and cell wall polysaccharide metabolism. Solyc09g008720.2, *ethylene receptor*. Solyc11g042560.2, *ethylene-responsive transcription factor*. Solyc02g080530.3, *peroxide*. Solyc07g026650.3, *1-aminocyclopropane-1-carboxylate oxidase* 5. Solyc07g055990.3, *xyloglucan endotransglucosylase/ hydrolase 7*. Solyc12g011030.3, *xyloglucan endotransglucosylase/ hydrolase 9.* Solyc08g077910.3, *expansin*. Solyc09g075330.3, *pectinase*. Solyc10g080210.2, *polygalacturonase-2*. Solyc09g075330.3, *pectinesterase.* XLOC_016662, XLOC_010878 and XLOC_033910 are predicted lncRNAs associated with the redox pathway. The dotted line indicates the speculation process

According to the gene expression analysis, 16 significantly differentially expressed genes **(**Additional file 6**)** were predicted to be related to fruit cracking in tomato, such as Solyc02g080530.3 (*Peroxide, POD*), Solyc01g008710.3 (*Mannan endo-1,4-beta-mannosidase, MAN*), Solyc08g077910.3 (*Expanded, EXP*), Solyc09g075330.3 (*Pectinesterase, PE*), Solyc07g055990.3 (*Xyloglucan endotransglucosylase-hydrolase 7, XTH7*), Solyc12g011030.2 (*Xyloglucan endotransglucosylase-hydrolase 9, XTH9*), Solyc10g080210.2 (*Polygalacturonase-2, PG2*), Solyc08g081010.2 (*Gamma-glutamylcysteine synthetase, gamma-GCS*), Solyc09g008720.2 (*Ethylene receptor, ER*), Solyc11g042560.2 (*Ethylene-responsive transcription factor 4, ERF4*) etc. **(**Table. 2 [47–70]). Hierarchical clustering analysis showed that the expression trends or levels of these genes in the two varieties were completely different after the irrigation treatment (Fig. 5a). For instance, the expression of *XTH7*, *XTH9*, *PE* and *POD* in the CR tomato showed a downward trend, while the expression in the CS tomato presented an upward trend. These genes play important roles in cell wall loosing and expansion. As disassembly of the fruit cell wall can influence fruit cracking [30], These plant cell-wall loosing genes may also play a key regulatory role in tomato fruit cracking. At the same time, we used Tomato Gene Expression Atlas (http://tea.solgenomics.net/expression_viewer/input) to verify the gene expression, and found that most differentially expressed genes in this experiment were expressed in tomato pericarp in red ripe stage. Among them, *GCS, MAN* and *PG,* the antioxidative genes and cell-wall degrading enzyme-associated genes showed the highest expression. Whereas high mRNA levels were never present in *ERF.* These might be because that the *ERF* is an upstream regulator [71], so it never presented a high expression in red ripe stage. The differences in gene expression in this experiment and Tomato Gene Expression Atlas might be due to different varieties, treatment and detection standards (Fig. 5b).

**Table 2:** Key genes related to tomato fruit cracking

Gene ID	Gene Function	Previous Studies
Solyc07g055990.3 Solyc12g011030.2 Solyc01g081060.2	xyloglucan endotransglucosylase-hydrolase 7 xyloglucan endotransglucosylase-hydrolase 9 xyloglucan endotransglucosylase-hydrolase 14 (XTH 7, XTH 9, XTH 14)	Park Y B [47] Jan A [48] He H [49]
Solyc10g080210.2 Solyc09g075330.3	Polygalacturonase-2 (PG 2) Pectinesterase (PE)	Tieman DM [50] Quesada MA [51]
Solyc08g077910.3	Expanded protein (EXP)	McQueen-Mason S [52]
Solyc01g008710.3	Mannan endo-1,4-beta-mannosidase (MAN)	Mo B [53] Stålbrand H [54] Mohammad I [55]
Solyc02g080530.3	Peroxide (POD)	Andrews J [56] Cordoba-Pedregosa C [57] Lin CC [58] Passardi F [59]
Solyc01g081250.3 Solyc08g081010.3	Glutathione-S-transferase (GST) Gamma-glutamylcysteine synthetase (gamma-GCS)	Jablonkai I [60] Kampranis SC [60] Marrs KA [61] Bartling D [62]
Solyc09g008720.2 Solyc11g042560.2	Ethylene receptor (ER) Ethylene-responsive transcription factor (ERF4)	Trainotti L [63] Ruther J [64] Hossain MA [65] Jeong SW [66] Kalaitzis P [67] Rose JK [68] Yoshida S [69]

Finally, we mapped a pathway diagram (Fig. 5c) of fruit cracking based on these differentially expressed lncRNAs, mRNAs and previous studies [71–77]. Within this pathway, *ERF*, *POD, PG* and *PE* play important roles. Previous researches suggests that ethylene influences fruit development and ripening (regulating cell wall-related *PG* and *EXP* gene expression) [71] and promotes programmed cell death of epithelial cells under ROS signalling [72]. Li et al. [73] showed that ARFs represent a point of cross-talk between ethylene and auxin signalling. Furthermore, auxin induces the production of ROS, and H_2_O_2_ decomposes polymers at the cell wall by producing ·OH [74]. Programmed cell death leads to a reduction in or loss of permeability of the plasma membrane, which in turn influences fruit cell activity, water absorption and cracking [75]. Simultaneously, the increase of auxin can promote the accumulation of H_2_O_2_ and the elongation of cells [76]. Furthermore, Rayle and Cleland [77] proposed the acid growth theory indicating that hydrogen ions may exert a purely chemical or physical effect, such as cleavage of acid-labile bonds on the wall, or they may activate normal enzymatic processes directly or indirectly, potentially leading to wall loosening. We analyzed the regulatory element in the promoter sequence of *PG, PE, EXP* and *XTH7* and found that there are Ethylene-responsive element and auxin-responsive element. Based on these findings, we speculate that the regulatory network of fruit cracking, especially the coexpression of cell wall-, redox-, and hormone-related mRNAs and their corresponding lncRNAs, influences fruit cracking.

### qRT-PCR validation of DEGs

Genes showing upregulated and the downregulated expression were randomly selected from the DEGs for qRT-PCR verification. The results of qRT-PCR revealed that most of these mRNAs shared similar expression tendencies to those indicated by the mRNA-Seq data, which can validate the reliability of our sequence data and our research results from the present study **(**Additional file 7**)**. The expression levels detected by the two methods were slightly different, which might have been due to the different detection ranges and sensitivities of the two detection methods. The comparison of the relative expression measured by qRT-PCR and RNA-seq was showed in Additional file 8, R^2^>0.7 confirmed the reliability of the RNA-Seq analysis results.

## Discussion

Tomato is one of the most popular commercial vegetables [78], however, its fruit shows high susceptibility to cracking [16, 31]. Cracks can occur throughout the fruit development stage during the ripening and post-harvest period [79, 80], which may cause serious economic losses. Different hypotheses have been presented to explain the occurrence of tomato fruit cracking. Previous studies have shown that rapid fruit swelling and fruit cracking are closely related [81]. Irregular temperatures or watering, especially a shift from a lower temperature to a much higher temperature or from extremely dry to very humid conditions, will lead to rapid swelling. The pressure of the rapidly expanding pulp on the peel may lead to fruit cracking [82]. Cell senescence and apoptosis also influence skin strength and water absorption, which can in turn affect fruit cracking [10]. In addition, a large differences between day and night temperatures can lead to the accumulation of carbohydrates [83]. Fruits with high levels of carbohydrates absorb more water, grow much faster and are more likely to crack [84]. In general, fruit cracking is a complex problem involving a mixture of genetics and the environment. Previous studies have suggested it is not a single gene but many genes that work together to regulate fruit cracking [31, 32].

### Cell wall polysaccharide metabolic

The DEG Solyc08g077910.3 encodes an *Expansin-like protein* that breaks down the hydrogen bonds between molecules in the cell wall macromolecular network to promote the depolymerization of the network, which can lead to relaxation of the cell wall [52]. In this experiment, the expression level of Solyc08g077910.3 was increased significantly after 8 h of irrigation (log2 fold-change = 7.13395) in CS tomato. The increased expression of the expansin-like gene can relax the cell wall and may influence fruit cracking.

Solyc07g055990.3 and Solyc12g011030.2 encode *xyloglucan endotransglucosylase/hydrolases 7 and 9*, respectively, which mediate the cleavage and polymerization of β-1,4-xyloglucan in the primary cell wall. Xyloglucan is usually fused to the cell wall, and its oligosaccharides determine tissue tension [47]. Jan [48] found that *OsXTH8* is involved in the cell wall modification process in rice and is highly expressed in the vascular bundle of the sheath and the young roots, in which the cells are rapidly elongated and differentiated. In addition, it can respond to gibberellin. He [49] found that *OsXTH5, OsXTH19, OsXTH20, OsXTH24* and *OsXTH28* play important roles in the elongation of rice peduncles and can respond to drought stress. These studies indicate that the *OsXTH* gene family plays an important role in the regulation of the structural function of rice cell walls. In this experiment, the expression levels of Solyc12g011030.2 and Solyc07g055990.3 in CS tomato showed an upward trend, while they showed a downward trend in the CR tomato **(**Fig. 5a**).** Simultaneously, the expression level in CS tomato was significantly higher than that in CR tomato. This illustrates that the CR tomato may exhibit a greater osmotic stress resistance ability with downregulation of the *XTH* gene that can strengthen the cell wall upon encountering water stress.

The DEG Solyc10g080210.2 (Polygalacturonase-2) can remove the methyl group from polygalacturonic acid; during tomato maturation, the degree of methylation decreases from 90% in the green ripen period to 35% in the red ripen period [50], which accelerates the degradation of the cell wall. In an antisense *PaPG1* transgenic study of strawberry, the expression level of *PG* was significantly inhibited, and the degree of fruit softening was significantly delayed [51].

### Redox processes

Previous studies have shown that peroxidase in the cell wall leads to cell wall sclerosis by causing cross-linking of cell wall components, thereby inhibiting cell elongation [56–58]. Peroxidase can also directly regulate plant cell elongation by controlling H_2_O_2_ levels [59]. Solyc02g080530.3 encodes peroxide, whose levels are significantly higher in CS tomato than in CR tomato. The expression of these genes in CS tomato fruits may increase cell wall hardness and hinder the elongation of the cell wall, which will lead to fruit cracking when water absorption swelling occurs. Solyc01g081250.3 encodes *glutathione-S-transferase* (*GST*). The *GST* superfamily enzymes exhibit multiple functions in plants. They are not only involved in primary metabolism and secondary metabolism [70], but they can also protect plants from oxidative damage and heterogeneous substances [60–62]. According to the data analysis, the gene expression of Solyc01g081250.3 in the CR tomato was significantly higher than that in the CS tomato after 0 h, 8 h and 30 h of irrigation treatment. Higher expression of *GST* in CR tomato can better maintain cell vigour and be beneficial to tomato fruits when coupled with water stress.

### Hormone-related

Previous research has shown that hormones can regulate the expression of cell wall-related genes. Trainotti [63] studied the expression of 32 genes related to cell wall synthesis and degradation. Their research showed that the expression of these genes in fleshy fruits can be inhibited by ethylene, while ethylene promotes the expression of these genes during fruit ripening and softening. At the same time, ethylene inhibits and promotes dual regulatory effects on the formation of plant secondary metabolites [64–66]; *TAPG1,* encoding a cell wall-degrading enzyme, can be induced by ethylene at the transcriptional level in tomato [67]; Rose [68] showed that ethylene regulates *LeEXP1*, which is specifically expressed only during fruit ripening. The pathway of ethylene biosynthesis in plants is the methionine cycle [85–87]. In this study, KEGG functional analysis of DEGs revealed significant enrichment in the methionine metabolic pathway. Solyc11g042560.2 encodes an ethylene receptor, while Solyc09g008720.2 encodes an ethylene-responsive transcription factor, and their expression levels are significantly upregulated after irrigation and are higher in CS tomato than in CR tomato.

### LncRNAs regulate tomato fruit cracking by coordinating gene expression in the hormone-redox-cell wall network

Liu’s research suggests that plants have gradually developed complex signalling pathways to cope with adverse environmental stimuli [8]. That is, plants perceive different stress signals from the circumstances in which they occur and then integrate these signals and respond to these different stresses by modulating the expression of related genes. Is it possible that cracking is also regulated by a complex network?

**Fig. 6:**
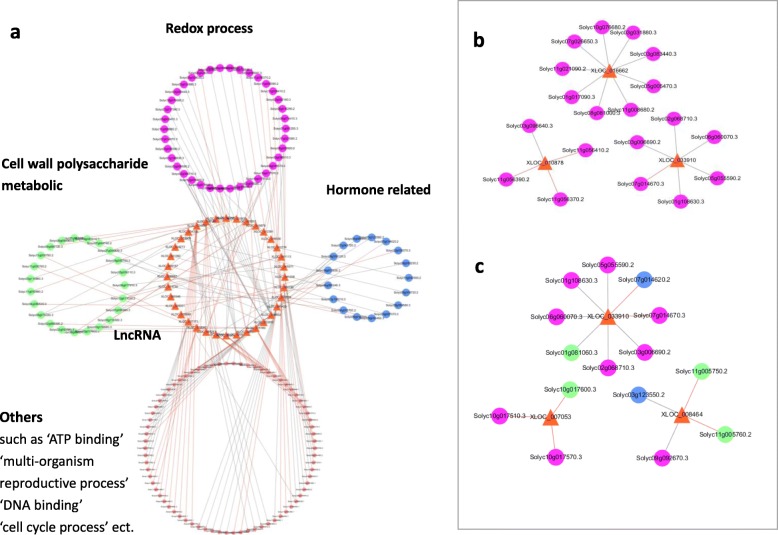
LncRNA-mRNA networks. The orange triangles represent lncRNAs, and the circles represent mRNAs (green: cell wall polysaccharide metabolism, yellow: redox process, blue: hormone-related, pink: other). The red edge represents the targeting mode of the lncRNA and the co-localized mRNA, and the grey edge represents co-expression. **a** The lncRNA-mRNA network influencing tomato fruit cracking. **b** LncRNAs that target the same kinds of mRNAs. **c** LncRNAs that target different kinds of mRNAs play an important role in the network

LncRNAs play important roles in epigenetic regulation, cell cycle regulation and many other activities. Here, we identified several lncRNAs that are involved in fruit cracking. Most lncRNAs are not annotated, and we do not know their functions. To predict the functions of these lncRNAs, we performed functional analysis of lncRNA-targeted mRNAs and constructed an lncRNA-mRNA network **(**Fig. 6**;** Additional file 9**)**. The results showed that the mRNAs in the network **(**Fig. 6a**)** were mainly enriched in the ‘oxidation-reduction process’, ‘oxidoreductase activity’, ‘hormone metabolic process’, ‘response to hormone stimulus’, ‘catalytic activity’, ‘cell wall organization’ and ‘external encapsulating structure’ categories. We classified the target genes into four categories (cell wall polysaccharide metabolism, oxidation-reduction processes, hormones and others) based on their functions and amounts.

Some lncRNAs specifically target functional mRNAs, and we can assume that the lncRNAs perform similar functions to their target mRNAs. For example, many of the target genes of XLOC_033910, XLOC_007053, and XLOC_008464 **(**Fig. 6b**)** are enriched in categories such as ‘dioxygenase activity’, ‘oxidation-reduction process’ and ‘oxidoreductase activity’, so we predicted their gene function as ‘redox regulation’.

Some lncRNAs are targets of significantly differentially expressed mRNAs with various functions. For example, for XLOC_033910 **(**Fig. 6c**)**, the target genes (Solyc06g060070.3,Solyc07g014670.3, Solyc05g055590.2, etc.) are enriched in the ‘oxidoreductase activity’ and ‘dioxygenase activity’ terms. XLOC_008464 also has other target genes, such as Solyc03g123550.1 (‘response to hormone stimulus’), and Solyc11g005750.2 (‘cell wall’). Previous studies [58–80] have shown that redox, hormone and cell wall terms are all very important factors that can influence fruit cracking, so we speculate that lncRNA XLOC_7053 may plays an important role in regulating tomato fruit cracking.

Liao’s research strongly supported *Ethylene-responsive factor 4* (*ERF4*) was associated with rind hardness and cracking resistance in watermelon fruits [88]. In our study, DEGs also included ethylene, as well as cell wall and redox related genes. Based on our research and previous research [67–69, 71–77], we mapped a “hormone-redox-cell wall” pathway diagram including lncRNA associated with fruit cracking.

## Conclusions

In this study, the key genes involved in the response to tomato cracking were identified by high-throughput sequencing, which has important significance for guiding the selection of new tomato varieties. We have also established an lncRNA-mRNA (hormone-redox-cell wall) network to learn about the precise regulation of fruit cracking by lncRNAs. To the best of our knowledge, this is the first discovery of the lncRNA-mRNA network involved in tomato fruit cracking.

## Methods

### Plant materials and sample collection

**Fig. 7:**
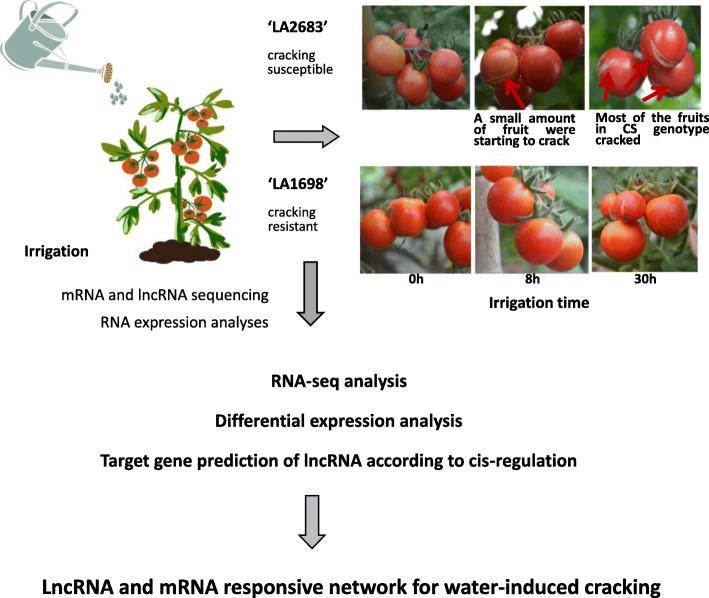
Experimental flow chart

RNA expression in 0 h, 8 h and 30 h irrigation-treated fruits from two contrasting tomato genotypes, ‘LA1698’ (cracking-resistant, CR) and ‘LA2683’ (cracking-susceptible, CS), was analysed by mRNA and lncRNA sequencing. ‘LA2683’ and ‘LA1698’ **(**Fig. 7**)** were both introduced by the Tomato Genetics Resource Centre (TGRC, University of California, Davis). The fruit cracking rates of ‘LA2683’ and ‘LA1698’ are 77.53 and 20.17% (Additional file 10), respectively. Both lines were selected and self-pollinated for more than 6 generations. All the seedlings were grown in 72-plug trays on 18 March 2016. On 28 April 2016, they were transplanted to the same greenhouse of the Kunshan Yuye Leaf Vegetable Base (31°95′E, 119°16′N), Suzhou, Jiangsu Province, China. The climate of this area belongs to the north subtropical south monsoon climate zone, with four distinct seasons and plenty of rainfall. The average annual temperature is 15.7 °C, and the annual average precipitation is 1094 mm according to China Weather Network (http://www.weather.com.cn/jiangsu). These two genotypes were planted side by side. Plant spacing followed a 30 × 50 × 100 cm pattern. Drip irrigation were adopted to guarantee the consistent of irrigation amount. Fertilization practices and pest control were those usually used by local growers. When fruits of the third cluster ripen, saturated irrigation based on previous researches [2, 16, 31] with slightly modificationwas adopted to induce fruit cracking. Specifically, plants were firstly irrigated using flood irrigation for 2 h. The field was essentially flooded with water which was allowed to totally soak into the soil. We then adopted drip irrigation to keep the soil saturated with water. After 0 h, 8 h and 30 h of irrigation treatment, for each genotype, treatment, one fruit were cut into four pieces from peduncle to blossom-end including exocarp and mesocarp without pulp. Each group has two repetitions. Samples of both genetypes were immediately frozen in liquid nitrogen and stored at − 80 °C. Sampling time is based on morphological observation. At 8 h, cracked fruits began to appear in cracking susceptible tomato ‘LA2683’ but not in cracking resistant tomato ‘LA1698’. At 30 h, the fruits of ‘LA2683’ severely cracked and fruits of ‘LA1698’ slightly cracked .

### RNA-seq analysis

Twelve fruit samples (2 genotypes, 3 time points, each with two replications) were collected and sequenced by Novogene, Beijing, China. RNA was extracted from the tomato pericarp, and qualified RNA samples were used to construct a cDNA library. Transcriptome sequencing was carried out on the Illumina HiSeq 2500 platform. Sequences showing low quality, linker contamination or a high unknown base (N) content were filtered from the raw reads obtained after sequencing. The filtered high-quality clean data were aligned to the ITAG4.0 reference genome using Hisat2 [89], and the transcriptome was assembled by using Cufflinks [90].

### Identification of mRNA and lncRNA

The transcripts that could be compared to known transcript data were identified as annotated mRNAs.

**Fig. 8:**
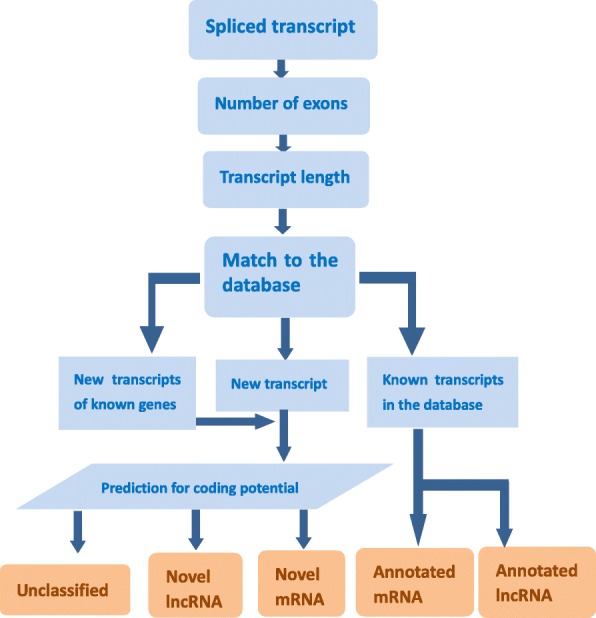
Transcript screening process

Then, the transcripts were screened according to the following criteria: (1) exon number ≥ 2 and (2) transcript length ≥ 200 bp. Additionally, (3) Transcripts that overlapped with the database annotation of the exon area according to Cuffcompare software were screened out. The lncRNAs overlapping with the exon region of a spliced transcript were included in the subsequent analysis. (4) The expression level of each transcript was calculated with Cuffquant, and transcripts with an FPKM≥0.5 were selected. (5) Two algorithms for evaluating protein-coding potential (CPC2 [91] and PFAM [92]) were used to predict the protein-coding potential of the remaining transcripts. Only when these two algorithms simultaneously indicated no protein-coding potential were the sequences considered to be predicted lncRNAs. Finally, the predicted lncRNAs were obtained. The transcripts showing the potential to encode proteins by “CPC2” and “PFAM” were identified as novel mRNAs **(**Fig. 8**)**.

### Analysis of mRNAs and lncRNAs

The mRNA and lncRNA abundance of the unigenes was normalized via the fragments per kilobase of exon model per million mapped reads (FPKM) approach. The log2 fold changes between two samples were tested statistically to determine whether the expression of an individual gene was significantly altered. A *Q*-value < 0.05 and |log2 fold-change| > 1 was considered to indicate a differentially expressed gene (DEG). Analyses of mRNA and lncRNA expression trends in tomato fruit after irrigation were then carried out. To understand the function of differentially expressed mRNAs, these mRNAs were further subjected to GO (Gene Ontology) and KEGG (Kyoto Encyclopedia of Genes and Genomes) analysis by using GOseq [93] and KOBAS [94] software, respectively.

### Feature analysis of lncRNAs and identification of lncRNA-mRNA pairs

Genomic characterization of the predicted lncRNAs was performed and compared with the mRNA results. The parameters for comparison to understand the genomic characteristics of the lncRNAs included the number of exons, ORF length, transcript nucleic acid length and sequence conservation between species.

LncRNAs mainly acts on their protein-coding target genes by cis-regulation or trans-regulation to achieve their regulatory functions. One of the functions of lncRNAs is cis-regulation of their neighbouring genes of the same allele; lncRNAs, which were located in less than 100 kb up/down stream of a gene, probably acted as cis-regulators. The up-stream lncRNAs having intersection of promoter or other cis-elements may regulate gene expression during or after transcription. The 3’UTR region or down-stream lncRNAs may perform other regulatory functions [95]. Another function of lncRNAs is the trans-regulation of co-expressed genes that are not adjacent to lncRNAs. For trans-regulation analysis, multiple repetitions are required for the result to be accurate and persuasive, samples with a value < 6 are not recommended [96, 97]. So in this study, lncRNAs by cis-regulation were analyzed. LncRNA within 100 kb upstream and downstream of the mRNA was identified as the lncRNA-mRNA pair and the function of lncRNAs was predicted through functional enrichment analysis of the cis target gene.

### qRT-PCR verification

Total RNA was extracted from tomato fruit by using the RNAprep Pure Plant Kit (Tiangen Biotech Co., Ltd. (Beijing, China)) following the manufacturer’s instructions. cDNA was generated using 5 μl of total RNA and abm’s 5X All-In-One MasterMix. qRT-PCR was performed in a CFX 96 Touch RT-PCR detection system (Bio-Rad, USA) with abm’s EvaGreen 2X qPCR MasterMix-Low ROX. Then, 9 DEGs were randomly selected from the DEGs to verify the RNA-seq results. Gene-specific primers were designed using Premier 5.0 [98] and *actin* as a reference gene [30]. The relative levels of gene expression were calculated using the 2^-ΔΔCT^ method [99] . The sequences of the primers are listed in Additional file 11**.**

## Supplementary information

**Additional file 1: Table S1.** LncRNAs in 12 tomato fruit libraries.

**Additional file 2: Table S2.** LncRNA-mRNA pairs were identified by cis-regulation.

**Additional file 3: Table S3.** Number of differentially expressed mRNAs and lncRNAs in different libraries.

**Additional file 4: Table S4.** GO analysis of DEGs between groups.

**Additional file 5: Table S5.** KEGG analysis of DEGs between groups.

**Additional file 6: Table S6.** Key genes related to tomato fruit cracking.

**Additional file 7: Figure S1.** Real-time PCR validation of high-throughput sequencing data. The x-axis represents the different time points of sampling, the left y-axis represents relative expression levels, and the right y-axis represents FPKM values. Blue bars represent data yielded by qRT-PCR, and red points represent data obtained by RNA sequencing. Different letters indicate significant differences (*P* < 0.05). (a) LA2683, (b) LA1698.

**Additional file 8: Figure S2.** The comparison of the relative expression measured by qRT-PCR and RNA-seq.

**Additional file 9: Table S7.** Data for the lncRNA-mRNA network.

**Additional file 10: Table S8.** Statistics for tomato cracking rate of ‘LA2683’ and ‘LA1698’.

**Additional file 11: Table S9.** Detailed primer sequences for qRT-PCR.

## Data Availability

The datasets generated during and/or analysed during the current study are included in this published article (and its supplementary information files). All RNA sequencing data from this study are available in the NCBI sequence read archive (SRA) under accession numbers: SAMN14409497, SAMN14409498, SAMN14409499, SAMN14409500, SAMN14409501, SAMN14409502, SAMN14409503, SAMN14409504, SAMN14409505, SAMN14409506, SAMN14409507 and SAMN14409508 (https://www.ncbi.nlm.nih.gov/sra/PRJNA613785).

## Abbreviations

CS: Cracking-susceptible
CR: Cracking-resistant
CS 0 h: Cracking-susceptible tomato fruit before irrigation (0 h)
CS 8 h: Cracking-susceptible tomato fruit after 8 h of irrigation
CS 30 h: Cracking-susceptible tomato fruit after 30 h of irrigation
CR 0 h: Cracking-resistant tomato fruit before irrigation (0 h)
CR 8 h: Cracking-resistant tomato fruit after 8 h of irrigation
CR 30 h: Cracking-resistant tomato fruit after 30 h of irrigation
DEGs: Differentially expressed genes
GO: Gene Ontology
KEGG: Kyoto Encyclopedia of Genes and Genomes
qRT-PCR: Quantitative real-time PCR
FPKM: Expected number of fragments per kilobase of transcript sequence per millions base pairs sequenced
