# Supplementary material for: LncRNA regulates tomato fruit cracking by coordinating gene expression via a hormone-redox-cell wall network
Source: BMC Plant Biol. 2020 Apr 15;20:162. doi: 10.1186/s12870-020-02373-9 (PMC7161180; doi:10.1186/s12870-020-02373-9)
Supplement: Supplementary file 7 — Additional file 7: Figure S1. Real-time PCR validation of high-throughput sequencing data. The x-axis represents the different time points of sampling, the left y-axis represents relative expression levels, and the right y-axis represents FPKM values. Blue bars represent data yielded by qRT-PCR, and red points represent data obtained by RNA sequencing. Different letters indicate significant differences (P < 0.05). (a) LA2683, (b) LA1698. [file 12870_2020_2373_MOESM7_ESM.docx]

a

b

c

b

a

c

b

a

b

b

a

b

b

a

c

b

a

c

b

a

a

b

b

b

b

a

c

b

a

c

b

a

c

b

a

a

a

b

a

c

b

b

b

a

a

b

b

b

c

b

a

c

b

a

b

a

RNA-seq

qRT-PCR
