# Supplementary figures and images for: LncRNA regulates tomato fruit cracking by coordinating gene expression via a hormone-redox-cell wall network
Source: BMC Plant Biol. 2020 Apr 15;20:162. doi: 10.1186/s12870-020-02373-9 (PMC7161180; doi:10.1186/s12870-020-02373-9)

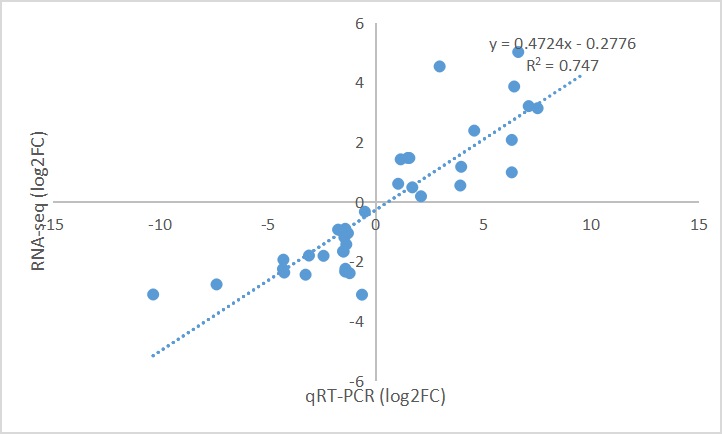

Supplement: Supplementary file 8 — Additional file 8: Figure S2. The comparison of the relative expression measured by qRT-PCR and RNA-seq. [file 12870_2020_2373_MOESM8_ESM.doc]
